# Clinical Surface Alteration of Reciprocating Nickel–Titanium Instruments Following Root Canal Preparation and Sterilization: A Three‐Dimensional FE‐SEM‐Based Analysis

**DOI:** 10.1155/ijod/7539273

**Published:** 2026-08-19

**Authors:** Nguyen-Huu-Phuoc Huynh, Hong-Hai Le, Nguyen-Minh-Hieu Tran, Hoang-Vinh Le, Huynh-Anh Bui, Thi-Thuy-Trang Huynh, Hoang-Lan-Anh Le, Phuong-Doan Phan, Nguyen-Tra-Mi Le, Thuan-Loc Tran, Trieu-Khang Pham, Cong-Minh Nguyen, Huynh-Thuy-Anh Bui, Thi-Bich-Van Tran, Quoc-Viet Lam, Quynh-Anh Lam, Dai-Phong Lam, Thi-Nguyet-Anh Nguyen, Thu-Tra Nguyen, Thi-Huong-Loan Pham, Gia-Khue Do, Van-Khoa Pham, Tran-Lan-Khue Pham, An-Tran Pham

**Affiliations:** ^1^ Faculty of Dentistry, University of Medicine and Pharmacy at Ho Chi Minh City, Ho Chi Minh City, Vietnam, hcmut.edu.vn; ^2^ Faculty of Odonto-Stomatology, University of Health Sciences, Vietnam National University, Ho Chi Minh City, Vietnam, vnu.edu.vn; ^3^ Faculty of Dentistry, Hong Bang International University, Ho Chi Minh City, Vietnam

**Keywords:** clinical instrumentation, dual-image FE-SEM, FE-SEM, NiTi, surface roughness

## Abstract

**Background:**

The study aims to evaluate the surface alteration of the reciprocating nickel–titanium (NiTi) instrument after clinical instrumentation.

**Methods:**

This clinical investigation evaluated surface changes of reciprocating NiTi instruments after routine endodontic use and sterilization. Eleven One RECI instruments, each used to prepare a single molar canal in one patient (11 patients, 11 canals, and 11 instruments), were evaluated. Maxillary and mandibular molars requiring primary treatment were instrumented using One RECI files under standardized clinical protocols. Following use‐and‐steam autoclave sterilization, instruments were analyzed using field‐emission scanning electron microscopy (FE‐SEM). Paired stereoscopic images obtained at standardized locations were reconstructed into three‐dimensional surface models using Mountains software (Digital Surf, Besancon, France). Areal surface roughness parameters, including Sa, Sq, and Sz, were calculated according to ISO 25178.

**Results:**

Clinical use resulted in increased Sa and Sq values at the cutting edge of the instruments, indicating measurable surface alteration after routine treatment and sterilization. These findings were consistent with trends previously reported under preclinical conditions.

**Conclusion:**

Routine clinical use and sterilization can induce detectable surface modifications in reciprocating NiTi instruments. Three‐dimensional FE‐SEM analysis provides a clinically relevant approach for assessing functional surface evolution under actual treatment conditions.

## 1. Introduction

Nickel–titanium (NiTi) instruments were continuously exposed to complex biomechanical and chemical conditions during clinical root canal preparation. While their superelasticity and flexibility enable effective shaping of curved canals, these same properties render their surface integrity highly sensitive to mechanical loading, frictional interaction with dentin, and chemical exposure to irrigants [1]. Surface condition plays a critical role in determining instrument performance as even minor microstructural alterations may influence cutting efficiency, stress distribution, and fatigue resistance during use [2].

During canal preparation, NiTi instruments experience repeated cycles of compression and tension while simultaneously interacting with dentin walls under irrigant‐rich conditions. This environment differs substantially from controlled laboratory simulations. In vivo instrumentation introduces biological fluids, anatomical variability, and operator‐dependent loading patterns that cannot be fully replicated in artificial models. These factors contribute to a dynamic surface evolution process that may affect the functional lifespan of instruments [3].

Previous investigations have primarily examined surface changes using simulated environments such as resin blocks or extracted teeth [3–5]. While such approaches allow for controlled comparison, they may not accurately reflect the tribological behavior of instruments in natural dentin. Resin materials differ significantly from dentin in hardness and thermal response, potentially altering frictional forces and wear mechanisms during instrumentation. The clinical relevance of surface findings derived from simulated models remains uncertain.

Besides mechanical interaction, chemical exposure during irrigation contributes to surface modification [6, 7]. Sodium hypochlorite is routinely used for canal disinfection and has been reported to influence the metallic surface of NiTi instruments [8]. Repeated contact with irrigants, particularly with mechanical stress, may promote corrosion or microtopographic alterations [8]. These changes may accumulate over time and influence the instrument’s durability.

Sterilization represents another clinically relevant factor. Instruments used in routine practice were subjected to autoclave cycles prior to reuse. Thermal exposure during sterilization may change oxide layers or induce microstructural adjustments [9]. Although sterilization is essential for infection control, its combined effect with clinical use on surface morphology remains insufficiently characterized under actual treatment conditions.

Surface irregularities such as grooves, pits, or microcracks can act as stress‐concentration sites and potentially contribute to fatigue failure [2]. The presence and evolution of such features are, therefore, important considerations when evaluating the instrument safety. Understanding how these irregularities develop in clinical settings may provide insights into the mechanisms underlying instrument degradation.

Advances in surface characterization have enabled a more detailed evaluation of metallic topography. Three‐dimensional analysis allows assessment of spatial surface features that cannot be fully described using linear measurements [10]. Areal surface parameters have been increasingly recognized as more representative of functional surface texture compared with profile‐based metrics [11]. Such approaches offer improved sensitivity for detecting subtle alterations induced by functional use.

Despite these developments, most available data remain derived from laboratory‐based investigations. There is limited information regarding how NiTi instrument surfaces behave following routine clinical use in natural teeth, where mechanical and chemical influences coexist [1, 3]. Evaluating surface evolution under actual treatment conditions is therefore essential for improving the translational relevance of research findings.

The aim of this study was to assess surface changes of reciprocating NiTi instruments following clinical root canal preparation and sterilization using three‐dimensional field‐emission scanning electron microscopy (FE‐SEM)‐based analysis.

## 2. Methods

This was a prospective observational clinical study using a paired, before‐and‐after measurement design. A total of 11 reciprocating One RECI instruments were evaluated, each used to prepare a single root canal in one molar of one patient (11 patients, 11 canals, and 11 instruments). Each instrument served as its own control, with surface roughness assessed at standardized locations before and after a single cycle of clinical instrumentation and sterilization. Reporting follows the STROBE statement for observational studies (Supporting Information).

This clinical investigation was designed to evaluate surface alterations of reciprocating NiTi instruments following routine endodontic preparation under standardized treatment conditions. All procedures were conducted in accordance with institutional ethical approval, and written informed consent was obtained from all participating patients prior to treatment.

Eligible teeth of the selected patients included maxillary and mandibular molars with fully developed apices requiring primary root canal therapy. Canals were included if they were negotiable with the size 8 stainless‐steel K‐file (Dentsply Sirona, Ballaigues, Switzerland) during initial exploration. Teeth presenting with previous endodontic treatment, internal or external resorption, severe calcification, or extensive structural damage were excluded to maintain procedural consistency and reduce anatomical variability.

Following administration of local anesthesia and rubber dam isolation, access cavities were prepared using a round bur to penetrate the pulp chamber and refined with an Endo‐Z bur (Dentsply Sirona, Ballaigues, Switzerland) to establish straight‐line access. Canal patency was verified and secured by gently advancing a size 8 K‐file until the file went through the apical foramen. The working length was determined by an electronic apex locator (ProPex Pixi, Dentsply Sirona, Ballaigues, Switzerland). Radiographic verification was performed to ensure the endodontic working length.

A glide path was established using size 10 and size 15 K‐files with gentle watch‐winding and balanced force movements. Irrigation was performed throughout the procedure using 3% sodium hypochlorite (Canal Pro, Coltene Whaledent, Altstätten, Switzerland) delivered via a needle positioned ~1–2 mm short of the working length. During instrumentation, 1 mL of 3% sodium hypochlorite was delivered after each ~3 mm of instrument advancement, with recapitulation between increments, so that the instrument remained in intermittent contact with the irrigant for the entire duration of canal preparation.

### 2.1. Reciprocating NiTi Instruments

One RECI file with a diameter of 0.25 mm, taper of 6%, and length of 21 mm (MicroMega, Besancon, France) was operated using an endodontic motor (Yoshi Ai‐Motor, Woodpecker, Guilin, China) in reciprocation mode according to the manufacturer’s recommended parameters. Each instrument was introduced into the canal using passive advancement with light apical pressure and controlled in‐and‐out movements. After every advancement of ~3 mm, the instrument was withdrawn from the canal and cleaned using a sterile gauze moistened with saline solution to remove accumulated debris. The canal was then irrigated with 1 mL of sodium hypochlorite and recapitulated using a size 10 K‐file to maintain patency and prevent blockage.

This sequence of advancement, withdrawal, cleaning, irrigation, and recapitulation was repeated incrementally until the full working length was achieved. Care was taken to avoid excessive apical pressure or prolonged engagement with canal walls to replicate routine clinical handling and minimize torsional stress.

Upon completion of canal preparation, the instrument used for each case was immediately removed and subjected to a standardized cleaning protocol. Gross debris was removed using sterile gauze, followed by rinsing with saline solution. The instruments were then packaged for sterilization.

Sterilization was performed using a steam autoclave operating at 134°C under a 30 psi pressure for 20 min. This procedure simulated routine clinical sterilization applied prior to potential instrument reuse. Each instrument was individually tracked throughout its clinical use‐and‐sterilization cycle to ensure an accurate correlation between treatment exposure and subsequent surface evaluation.

Surface analysis was conducted using FE‐SEM. FE‐SEM was selected because it provides high spatial resolution and an extended depth of field suitable for imaging fine metallic surface features and because paired stereoscopic FE‐SEM images can be reconstructed into quantitative three‐dimensional surface models. Prior to imaging, instruments were mounted in custom stabilization molds designed to ensure reproducible orientation across imaging sessions. Correct positioning was verified under optical magnification before placement into the SEM chamber [4].

Surface imaging was performed at standardized locations along the cutting edge and flute, ~3 mm from the instrument tip [4]. At each location, two SEM images were obtained using a stereoscopic imaging approach. The first image was captured with the stage positioned at a 0° tilt to provide a perpendicular view of the surface. The second image was obtained after adjusting the stage to a 5° tilt while maintaining identical lateral positioning. This ensured that both images corresponded to the same surface region.

The paired images were saved in high‐resolution format and imported into Mountains software for three‐dimensional reconstruction. The reconstruction process involved the alignment of corresponding surface features between the two images using triangulation principles. Spatial coordinates were calculated to generate a digital surface model representing microtopographic features.

Noise reduction filters were applied to remove isolated imaging artifacts while preserving true surface structures. Form removal was performed to eliminate the curvature associated with the cylindrical geometry of the instrument. A leveling operation based on a least‐squares reference plane standardized the orientation across samples.

From the reconstructed three‐dimensional datasets, areal surface roughness parameters were calculated under ISO 25,178 definitions. These included Sa (arithmetic mean height), Sq (root‐mean‐square height), and Sz (maximum peak‐to‐valley height).

Through this workflow, qualitative SEM observations were converted into quantitative three‐dimensional measurements, enabling the evaluation of surface changes associated with clinical instrumentation and sterilization.

## 3. Statistical Analysis

All analyses were performed at a significance level of *p*  < 0.05. The distribution of each surface roughness parameter was assessed using the Shapiro–Wilk test. For normally distributed parameters, before‐and‐after comparisons were conducted using the paired *t*‐test, and the results are reported as mean differences with 95% confidence intervals. For parameters that deviated significantly from normality, the nonparametric Wilcoxon signed‐rank test was applied, and results were reported as median differences with 95% confidence intervals. The choice of the test was therefore determined individually for each parameter based on its distribution.

## 4. Results

**Figure 1:**
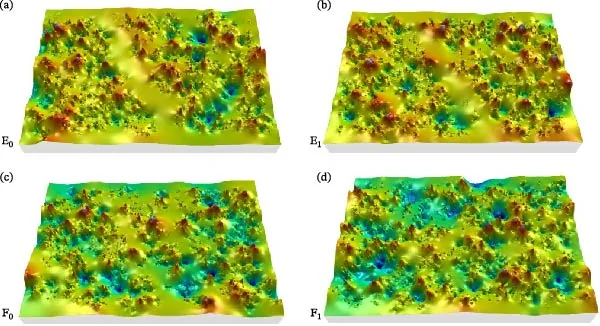
The representative color‐coded three‐dimensional surface reconstructions of a One RECI reciprocating instrument were obtained at the cutting edge and the flute ~3 mm from the instrument tip, before ([a] E_0_ and [c] F_0_) and after ([b] E_1_ and [d] F_1_) one clinical use‐and‐sterilization cycle. Three‐dimensional models were reconstructed from paired stereoscopic images (0° and 5° stage tilt) using the Mountains software (Digital Surf, Besancon, France) and processed by noise reduction, form removal, and least‐squares leveling. Color scales represent surface height (µm), with warmer colors indicating peaks and cooler colors indicating valleys; areal roughness parameters (Sa, Sq, and Sz) were computed from these reconstructions according to ISO 25178.

Three‐dimensional FE‐SEM data with dual images reconstructed using Mountains software illustrated noticeable changes in the surface morphology after clinical instrumentation. Eleven patients were enrolled, and 11 molar canals were instrumented using 11 One RECI instruments; the sample comprised 6 mandibular and 5 maxillary molars. All 11 instruments were successfully imaged before and after the clinical cycle with no missing data. Each instrument was examined at two locations: the cutting edge and the flute. At each location, FE‐SEM images were captured before and after one clinical use cycle to quantify surface wear. Surface roughness values before and after the clinical instrumentation are summarized in Table 1, and the corresponding paired comparisons are presented in Table 2. Representative reconstructed three‐dimensional surface models are shown in Figure 1.

**Table 1:** The surface roughness of the experimental groups before and after the clinical instrumentation with the One RECI instrument.

Position	Surface roughness	Arithmetic mean	95% CI for the arithmetic mean	Standard deviation	Median	95% CI for the median	Normal distribution (*p*)
Edge	SaE_0_	14.3391	13.3406 to 15.3376	1.4862	14.7800	12.7907 to 15.4350	Accept (0.7560)
	SaE_1_	16.6764	14.6247 to 18.7281	3.0540	15.6300	14.9233 to 17.8051	Reject (0.0204 ^∗^)
	SqE_0_	20.3836	19.3896 to 21.3777	1.4796	20.5900	18.8920 to 21.4818	Accept (0.6405)
	SqE_1_	23.2455	20.8023 to 25.6886	3.6367	21.9700	21.3461 to 25.0535	Reject (0.0054 ^∗^)
	SzE_0_	255.6273	242.6942 to 268.5603	19.2511	253.4800	241.3026 to 271.5390	Accept (0.7571)
	SzE_1_	268.6455	259.4453 to 277.8456	13.6945	270.1700	257.8966 to 281.4582	Accept (0.8256)
Flute	SaF_0_	15.4118	15.0702 to 15.7534	0.5084	15.4300	15.0507 to 15.8854	Accept (0.9942)
	SaF_1_	16.8473	15.4592 to 18.2353	2.0662	15.9300	15.3924 to 17.9504	Accept (0.0560)
	SqF_0_	21.7827	21.1893 to 22.3761	0.8833	21.8600	21.1896 to 22.3709	Accept (0.8113)
	SqF_1_	23.4936	21.5018 to 25.4855	2.9649	22.9500	21.2258 to 25.2977	Accept (0.1315)
	SzF_0_	265.0691	249.0415 to 281.0967	23.8573	263.6200	248.3970 to 275.0389	Accept (0.2455)
	SzF_1_	269.3627	259.5428 to 279.1826	14.6171	270.3600	259.9911 to 284.7599	Accept (0.7494)

*Note:* 0: before clinical instrumentation; 1: after clinical instrumentation.  ^∗^ *p* < 0.05: Shapiro–Wilk test for normal distribution.

Abbreviations: E, edge; F, flute.

**Table 2:** The differences in surface roughness, Sa, Sq, and Sz, between the before‐and‐after clinical instrumentation at the edge and the flute of the One RECI instrument.

Surface roughness	Median difference	95% CI	Mean difference	95% CI of difference	*p*
SaE_0_, SaE_1_	1.2200	0.3900 to 4.9050	N/A	N/A	0.0244 ^∗^
SqE_0_, SqE_1_	2.0975	0.3900 to 6.0500	N/A	N/A	0.0098 ^∗^
SzE_0_, SzE_1_	N/A	N/A	13.0182	−3.4956 to 29.5320	0.1095⁑
SaF_0_, SaF_1_	N/A	N/A	1.4355	−0.03121 to 2.9021	0.0542⁑
SqF_0_, SqF_1_	N/A	N/A	1.7109	−0.4451 to 3.8669	0.1075⁑
SzF_0_, SzF_1_	N/A	N/A	4.2936	−12.4317 to 21.0189	0.5800⁑

*Note:* 0: before clinical instrumentation; 1: after clinical instrumentation.

Abbreviations: E, edge; F, flute; N/A: not applicable.

^∗^ *p*  < 0.05: Wilcoxon test.

⁑*p*  > 0.05: paired *t*‐test.

There are two groups that have been distributed nonnormally at the edge of the instrument after clinical instrumentation.

There are two significant differences in surface roughness between Sa and Sq before‐and‐after clinical instrumentation at the edge of the One RECI instrument.

## 5. Discussion

The results of the present study show that there are increases in Sa and Sq values at the edge of the One RECI instrument after clinical use on the patient. These results are like those of the previous study in the preclinical environment [4].

The present clinical investigation aimed to evaluate how reciprocating NiTi instruments behave at the surface level after routine endodontic use under actual treatment conditions. Unlike laboratory simulations, clinical instrumentation exposes NiTi instruments to a combination of mechanical loading, dentin friction, chemical irrigants, and thermal sterilization cycles. These factors interact simultaneously and may contribute to progressive surface modification. Understanding this surface evolution is important because microtopographic irregularities have been associated with fatigue crack initiation and eventual mechanical failure [2].

During root canal preparation, NiTi instruments are subjected to cyclic tension–compression stresses as they navigate curved canals while simultaneously engaging the dentin walls. This mechanical interaction can induce surface wear or plastic deformation [1]. Previous studies have reported that surface defects such as pits, grooves, or microcracks may develop during instrumentation and act as stress‐concentration sites that reduce fatigue resistance [3]. The findings of the present study support the concept that even routine clinical use can lead to measurable changes in the surface morphology.

Chemical exposure represents an additional factor influencing surface integrity. Sodium hypochlorite, widely used for irrigation, has been shown to interact with NiTi alloys and potentially contribute to surface alteration [8]. The coexistence of mechanical stress and chemical exposure may speed up localized degradation processes. Such interactions are difficult to replicate in simulated environments and highlight the importance of evaluating instruments under clinical conditions. Atomic force microscopy studies have similarly demonstrated that sodium hypochlorite can induce surface pitting and roughening of NiTi instruments [12–14].

Sterilization further contributes to the surface modification. Steam autoclaving has been reported to influence the oxide layer of NiTi instruments and may induce microstructural changes following repeated cycles [9]. While sterilization is essential for infection control, its cumulative effect alongside clinical use may contribute to subtle topographic alterations. Incorporating sterilization into the present workflow therefore enhances the clinical relevance of the findings.

It should be noted that the present design evaluated the combined effect of mechanical instrumentation, chemical irrigation, and steam sterilization as a single clinical cycle. Accordingly, the observed surface alterations represent the net outcome of this composite exposure, and the individual contribution of sterilization relative to mechanical use cannot be isolated from the present data.

Many previous investigations assessing NiTi surface characteristics have relied on simulated models using resin blocks or extracted teeth [3, 4]. Although these models offer standardized conditions, they differ from natural dentin in hardness and thermal behavior. Resin materials may soften under frictional heat, potentially altering wear patterns and influencing the instrument behavior. Results derived from simulated environments may not fully represent the clinical performance.

Natural dentin presents a more complex tribological environment, where anatomical variability and biological fluids may influence frictional interactions. These factors may contribute to differences in surface evolution compared with those in laboratory simulations. Evaluating instruments following their use in natural teeth, therefore, provides valuable insight into real‐world performance.

Surface integrity plays a critical role in determining the mechanical behavior. Microdefects formed during use may act as initiation sites for fatigue cracks under cyclic loading [2]. Even minor changes in the surface texture can influence stress distribution and potentially affect resistance to fatigue failure. Modern manufacturing processes such as heat treatment or electropolishing have been introduced to improve flexibility and fatigue resistance [15]. However, these approaches do not eliminate surface alterations induced by clinical use.

The One RECI instrument is intended for a single use. The increase in edge roughness (Sa from ~14.3–16.7 µm; Sq from ~20.4–23.2 µm) detected after a single clinical cycle therefore indicates that measurable surface evolution occurs even within the manufacturer‐intended lifespan of the instrument. Mechanistically, such surface irregularities may serve as stress‐concentration sites favoring fatigue crack initiation. However, as the present study did not directly assess cyclic fatigue or torsional resistance, the precise clinical threshold at which this magnitude of surface change becomes mechanically consequential cannot be determined from these data and warrants dedicated mechanical testing.

Traditional qualitative FE‐SEM analysis has been widely used to identify surface defects [16]. While effective for visualization, qualitative assessment alone may not detect subtle microtopographic changes. Quantitative surface analysis allows for a more comprehensive evaluation of these alterations. Areal surface parameters provide information regarding the height distribution across a defined region and are considered more representative of functional topography than linear profile measurements [10, 11].

Compared with alternative surface‐characterization approaches, qualitative SEM permits visualization of surface defects but does not yield quantitative height data, while contact and optical profilometry provide quantitative measurements but may be limited in lateral resolution or by the curved geometry of endodontic instruments. Stereoscopic three‐dimensional FE‐SEM reconstruction combines high‐resolution imaging with quantitative areal parameters and was therefore considered suitable for detecting subtle clinically induced surface alterations.

Using three‐dimensional reconstruction further enhances the ability to assess the surface morphology. Stereoscopic FE‐SEM imaging enables the generation of digital surface models that incorporate depth information [17]. This approach allows a transition from descriptive observation to quantitative analysis and supports a more detailed evaluation of surface changes following clinical use.

Previous studies have demonstrated that different NiTi systems may exhibit distinct surface responses to mechanical and chemical exposures [3, 4, 6, 8]. Variations in alloy treatment, cross‐sectional design, and manufacturing processes may influence how instruments interact with dentin and respond to clinical stresses [15]. Such variability reinforces the importance of evaluating instruments under conditions that closely resemble routine practice.

The findings of the present study suggest that the surface morphology of reciprocating NiTi instruments is dynamically influenced by clinical use and sterilization. This observation aligns with the broader understanding that surface integrity develops during functional use rather than remaining static. Monitoring these changes may contribute to an improved understanding of instrument durability.

Importantly, the study emphasizes the need to interpret laboratory findings cautiously. Simulated environments provide valuable mechanistic insights but may not fully capture the complexity of clinical use. Instruments operating in vivo are exposed to biological fluids, anatomical variations, and operator‐dependent loading patterns that may influence surface evolution [1].

The functional importance of surface texture has been widely recognized in materials science [11]. In dental applications, three‐dimensional analysis has been used to assess surface modifications induced by mechanical preparation or chemical exposure [18]. Extending such approaches to the clinical evaluation of NiTi instruments enhances their translational relevance.

Overall, the results show that routine clinical use combined with sterilization may influence the surface characteristics of reciprocating NiTi instruments. While modern manufacturing processes improve mechanical performance, surface integrity remains susceptible to functional loading and environmental interactions. Future studies incorporating longitudinal evaluation across multiple clinical uses may further clarify the relationship between surface alteration and fatigue resistance.

The present findings pertain to the specific protocol employed, namely, steam autoclave sterilization and reciprocating motion. Alternative sterilization approaches and other kinematic modes reported in the literature may affect surface morphology differently, and a direct comparison of these variables was beyond the scope of this study.

## 6. Limitations

Several limitations should be considered when interpreting these findings. First, the study evaluated a single reciprocating system (One RECI); comparison with other reciprocating or rotary systems would be required to determine whether the observed surface alterations are system‐specific or generalizable. Second, canal curvature was not quantified (e.g., by Schneider’s method), so anatomical variability represents an uncontrolled variable, although inclusion was restricted to molars negotiable with a size 8 K‐file to improve consistency. Third, the design assessed the composite effect of one complete clinical cycle (mechanical instrumentation, irrigation, and steam sterilization) and therefore cannot isolate the individual contribution of sterilization from that of clinical use. Fourth, an a priori sample‐size calculation was not performed; while the primary edge parameters reached statistical significance, nonsignificant results for other parameters should be interpreted with caution given the possibility of a Type II error. Fifth, surface chemistry was not assessed; complementary techniques such as energy‐dispersive X‐ray (EDX) analysis [19], together with direct mechanical testing of cyclic fatigue and microhardness, would provide a more complete characterization of clinically induced instrument degradation. These limitations define clear directions for future research.

## Author Contributions

**Huynh-Thuy-Anh Bui** **, Huynh-Anh Bui, Thi-Thuy-Trang Huynh, Hoang-Lan-Anh Le, Hoang-Vinh Le, Nguyen-Tra-Mi Le, Cong-Minh Nguyen, Thi-Nguyet-Anh Nguyen, Thu-Tra Nguyen, Thi-Huong-Loan Pham, Dai-Phong Lam, and Phuong-Doan Phan**: data curation, formal analysis, supervision, validation, writing – review and editing. **Nguyen-Huu-Phuoc Huynh**: data curation, formal analysis, methodology, project administration, writing – original draft, writing – review and editing. **Van-Khoa Pham**: conceptualization, data curation, formal analysis, funding acquisition, investigation, methodology, project administration, resources, software, supervision, validation, visualization, writing – original draft, writing – review and editing. **Quoc-Viet Lam**: data curation, formal analysis, investigation, supervision, validation, visualization, writing – review and editing. **Hong-Hai Le**: data curation, investigation, supervision, writing – original draft, writing – review and editing. **An-Tran Pham**: conceptualization, data curation, formal analysis, investigation, methodology, project administration, supervision, validation, visualization, writing – original draft, writing – review and editing. **Trieu-Khang Pham**: conceptualization, data curation, formal analysis, investigation, methodology, supervision, validation, visualization, writing – original draft, writing – review and editing. **Tran-Lan-Khue Pham**: conceptualization, data curation, formal analysis, investigation, methodology, project administration, supervision, validation, visualization, writing – original draft, writing – review and editing. **Thi-Bich-Van Tran**: data curation, formal analysis, investigation, supervision, validation, visualization, writing – review and editing. **Thuan-Loc Tran**: data curation, formal analysis, supervision, validation, writing – review and editing.

## Funding

The authors received no specific funding for this work.

## Disclosure

All authors have read and approved the final version of the manuscript. The corresponding author had full access to all the data in this study and takes complete responsibility for the integrity of the data and the accuracy of the data analysis.

## Ethics Statement

This study was approved by the Research Ethics Committee of the University of Medicine and Pharmacy at Ho Chi Minh City, Vietnam (Approval Number 940/HĐĐĐ‐ĐHYD). The study was conducted in vivo, with instrumentation performed during routine primary root canal treatment on patients; no extracted teeth were used. Written informed consent was obtained from all participants prior to treatment, including consent for the use of the endodontic instruments used during their root canal therapy for subsequent surface analysis. All procedures performed in the study involving human participants were in accordance with the ethical standards of the institutional and/or national research committee and with the 1964 Helsinki Declaration and its later amendments or comparable ethical standards.

## Conflicts of Interest

The authors declare no conflicts of interest.

## Supporting Information

Additional supporting information can be found online in the Supporting Information section.

## Supporting information


**Supporting Information** The STROBE_Checklist was supplied in the supporting information.

## Data Availability

The authors confirm that the data supporting the findings of this study are available in the article. Additional raw datasets are available from the corresponding author upon reasonable request.
