# Supplementary material for: Clinical Surface Alteration of Reciprocating Nickel–Titanium Instruments Following Root Canal Preparation and Sterilization: A Three‐Dimensional FE‐SEM‐Based Analysis
Source: Int J Dent. 2026 Aug 19;2026:7539273. doi: 10.1155/ijod/7539273 (PMC13488369; doi:10.1155/ijod/7539273)
Supplement: Supplementary file 1 [file IJOD-2026-7539273-s001.docx]

**STROBE Statement — Checklist for Cross-Sectional Studies**

*Manuscript: Clinical Surface Alteration of Reciprocating Nickel–Titanium Instruments Following Root Canal Preparation and Sterilization: A Three-Dimensional FE-SEM-Based Analysis*

The page/section references below correspond to the revised manuscript. Items shown in red require the authors to insert final page numbers after pagination or to add content that is not yet present in the manuscript (notably the sample size and participant numbers).

| **No.** | **STROBE Item** | **Recommendation** | **Reported in manuscript (section / [page])** |
| --- | --- | --- | --- |
| **Title and Abstract** | | | |
| **1** | Title and abstract | (a) Indicate the study’s design with a commonly used term in the title or the abstract. (b) Provide in the abstract an informative and balanced summary of what was done and what was found. | Title; Abstract [p. 1].  Add design term: revised Methods now states “prospective observational clinical study, paired before-and-after design.” Recommend echoing this in the title/abstract. |
| **Introduction** | | | |
| **2** | Background / rationale | Explain the scientific background and rationale for the investigation being reported. | Introduction, paragraphs 1–8 [p. 2–3]. |
| **3** | Objectives | State specific objectives, including any prespecified hypotheses. | Introduction, final paragraph: aim to assess surface changes of reciprocating NiTi instruments after clinical preparation and sterilization using 3D FE-SEM [p. 3]. |
| **Methods** | | | |
| **4** | Study design | Present key elements of study design early in the paper. | Methods, opening (revised): prospective observational clinical study, paired before-and-after design, each instrument its own control; STROBE-compliant reporting [p. 3]. |
| **5** | Setting | Describe the setting, locations, and relevant dates, including periods of recruitment, exposure, follow-up, and data collection. | Setting: Faculty of Dentistry, University of Medicine and Pharmacy at Ho Chi Minh City; ethics approval 940/HĐĐĐ-ĐHYD [Methods; Ethics, p. 3, p. 6].  Add recruitment / data-collection dates (months, year) — not currently stated. |
| **6** | Participants | (a) Give the eligibility criteria, and the sources and methods of selection of participants. | Eligibility criteria stated: maxillary/mandibular molars, fully developed apices, primary RCT, canals negotiable to size 8 K-file; exclusion criteria listed [Methods, p. 3].  Sample: 11 patients, 11 molar canals, 11 One RECI instruments (one instrument per canal per patient) [Methods/Results]. |
| **7** | Variables | Clearly define all outcomes, exposures, predictors, potential confounders, and effect modifiers. | Outcomes: areal surface roughness Sa, Sq, Sz (ISO 25178), measured at edge and flute, before vs after one clinical-use-plus-sterilization cycle [Methods, p. 4]. |
| **8** | Data sources / measurement | For each variable of interest, give sources of data and details of methods of assessment (measurement). Describe comparability of assessment methods if there is more than one group. | FE-SEM stereoscopic imaging (0° and 5° tilt) at standardized locations ~3 mm from tip; 3D reconstruction in Mountains software; noise reduction, form removal, least-squares leveling; ISO 25178 parameters [Methods, p. 3–4]. |
| **9** | Bias | Describe any efforts to address potential sources of bias. | Standardized clinical protocol, individual instrument tracking, reproducible mounting/orientation, fixed imaging location [Methods, p. 3–4].  Curvature not quantified (acknowledged in Limitations); consider stating operator standardization / blinding of measurement. |
| **10** | Study size | Explain how the study size was arrived at. | Eleven instruments (n = 11), each used in one molar canal of one patient, were analyzed before and after one clinical-use-plus-sterilization cycle [Methods/Results].  No a priori sample-size calculation was performed; this convenience sample and the absence of a power calculation are acknowledged in the Limitations (revised Discussion). |
| **11** | Quantitative variables | Explain how quantitative variables were handled in the analyses. If applicable, describe which groupings were chosen and why. | Sa, Sq, Sz treated as continuous; compared by location (edge/flute) and time (before/after). Statistical Analysis subsection (revised) [Methods, p. 4]. |
| **12** | Statistical methods | (a) Describe all statistical methods, including those used to control for confounding. (b) Describe any methods used to examine subgroups and interactions. (c) Explain how missing data were addressed. (d)–(e) cross-sectional specifics. | Statistical Analysis subsection (revised): Shapiro–Wilk normality test; paired t-test for normal parameters, Wilcoxon signed-rank test for non-normal parameters; significance P < 0.05; results as mean/median differences with 95% CI [Methods, p. 4; Tables 1–2]. |
| **Results** | | | |
| **13** | Participants | (a) Report numbers of individuals at each stage of study. (b) Give reasons for non-participation. (c) Consider use of a flow diagram. | Eleven patients were enrolled; 11 molar canals (6 mandibular, 5 maxillary) were instrumented with 11 One RECI instruments, and all 11 instruments were imaged before and after the clinical cycle (no dropouts). State this explicitly in Results [Results]. |
| **14** | Descriptive data | (a) Give characteristics of study participants and information on exposures and potential confounders. (b) Indicate number of participants with missing data. | n = 11 (11 patients, 11 molar canals, 11 instruments), comprising 6 mandibular and 5 maxillary molars. No missing data: all 11 instruments were imaged both before and after the clinical cycle [Results]. |
| **15** | Outcome data | Report numbers of outcome events or summary measures (cross-sectional). | Table 1: arithmetic mean, SD, median, 95% CI and Shapiro–Wilk P for Sa/Sq/Sz at edge and flute, before/after [Results, p. 4–5]. |
| **16** | Main results | (a) Give unadjusted estimates and, if applicable, confounder-adjusted estimates and their precision (e.g., 95% CI). (b) Report category boundaries when continuous variables were categorized. (c) Translate relative risk into absolute risk for a meaningful time period if relevant. | Table 2: median/mean differences with 95% CI and P values; significant increases in SaE and SqE at the edge (P = 0.0244 and P = 0.0098). Narrative statement of significant edge differences [Results, p. 5–6]. |
| **17** | Other analyses | Report other analyses done—e.g., analyses of subgroups and interactions, and sensitivity analyses. | Per-location analysis (edge vs flute) reported in Tables 1–2. No additional subgroup/sensitivity analyses were performed [Results, p. 4–6]. |
| **Discussion** | | | |
| **18** | Key results | Summarise key results with reference to study objectives. | Discussion, opening: increases in Sa and Sq at the edge after clinical use, consistent with preclinical findings [p. 6]. |
| **19** | Limitations | Discuss limitations of the study, taking into account sources of potential bias or imprecision. Discuss both direction and magnitude of any potential bias. | Dedicated Limitations paragraph (revised): single system, unquantified curvature, composite clinical cycle (cannot isolate sterilization), no a priori sample-size calculation / Type II error, no surface-chemistry or mechanical testing [Discussion, p. 7–8]. |
| **20** | Interpretation | Give a cautious overall interpretation of results considering objectives, limitations, multiplicity of analyses, results from similar studies, and other relevant evidence. | Discussion: mechanistic interpretation of roughness increase relative to single-use indication and fatigue-crack initiation, with explicit caution that fatigue/torsional resistance were not measured; comparison with alternative methods and prior literature [revised Discussion, p. 6–8]. |
| **21** | Generalisability | Discuss the generalisability (external validity) of the study results. | Discussion: findings pertain to the specific One RECI system and the steam-sterilization/reciprocation protocol employed; generalization to other systems/protocols identified as future work [revised Discussion, p. 7–8]. |
| **Other Information** | | | |
| **22** | Funding | Give the source of funding and the role of the funders for the present study and, if applicable, for the original study on which the present article is based. | Funding statement (revised): “This study did not receive any specific funding.” [p. 8]. |

*Note: An Explanation and Elaboration article discusses each checklist item and is available at https://www.strobe-statement.org. The STROBE checklist is distributed under a Creative Commons Attribution license.*
